# GPs who volunteer to be first responders for out-of-hospital cardiac arrest: A qualitative study

**DOI:** 10.1080/13814788.2019.1681194

**Published:** 2019-11-05

**Authors:** Tomas Barry, Suzanne Guerin, Mary Headon, Gerard Bury

**Affiliations:** a UCD Centre for Emergency Medical Science, School of Medicine, Health Sciences Centre, University College Dublin, Dublin, Ireland;; b School of Psychology, University College Dublin, Dublin, Ireland

**Keywords:** Primary healthcare, general practice, out-of-hospital cardiac arrest, qualitative research, emergency responders

## Abstract

**Background:** Out-of-hospital cardiac arrest (OHCA) is a major cause of premature mortality. Survival is possible when timely cardiopulmonary resuscitation and defibrillation are available in the community. GPs are well placed to provide early OHCA care and significantly increased rates of survival are achieved when GPs participate in resuscitation. A novel project alerts volunteer GP first responders to nearby OHCAs in Ireland.

**Objectives:** To explore the reasons why GPs volunteer to be OHCA first responders and their experience of participation.

**Methods:** A qualitative study involving in-depth, semi-structured interviews followed by thematic analysis was undertaken in 2017/18. Fourteen GPs from differing geographical areas in Ireland, who volunteered as OHCA first-responders were recruited to participate by purposive methods.

Results: GP participation in OHCA voluntary first response was understood as a function of GPs relationship to the community, their ability to manage competing demands in their personal and professional lives and also specific participatory gains. GPs expressed both altruistic motivations and a sense of obligation. GPs described a complex, multifaceted role in providing OHCA first response; they derived an inherent sense of satisfaction in delivering potentially life-saving interventions but also in the provision of holistic, compassionate end-of-life care for patients and their families. Participation was not without psychosocial risk for GPs.

**Conclusion:** GPs volunteer to provide early OHCA emergency care because of their relationship to the community. Care provided is complex and includes both resuscitation and end-of-life care.

KEY MESSAGES

- GPs contribute significantly to survival from cardiac arrest. In Ireland, emergency medical services now alert volunteer GP first responders.
- GPs volunteer for this role because of their relationship to the community.
- GP first responders provide holistic cardiac arrest care including basic life support, team working with paramedics, critical decision-making and psychosocial care for bystanders and family members.

## Introduction

Out-of-hospital cardiac arrest (OHCA) has a high mortality; approximately 275,000 people have resuscitation attempted annually in Europe, 10% survive [1]. Resuscitation from OHCA is largely determined by the availability of cardiopulmonary resuscitation (CPR) and defibrillation in its immediate aftermath. Each minute delay reduces survival by approximately 10% [2]. General practitioners (GPs) are well placed to deliver early community based cardiac arrest care provided they are appropriately trained, equipped, and willing to participate. When GPs participate in OHCA resuscitation, markedly increased patient survival has been reported [3–5].

The Republic of Ireland experiences over 2000 OHCAs per year where resuscitation is attempted and has approximately 3000 GPs [6–8]. The MERIT (medical emergency responders integration and training) project established in 2005 [9] supports the training and equipment of GPs for cardiac arrest management; over 500 defibrillators (AEDs) have been provided to individual practices and 50% of all Irish GPs have attended emergency care training [10]. Over 10 years, half of all participating practices and two thirds of rural practices managed one or more cardiac arrest resuscitation attempt(s) and Irish GPs were likely involved in the resuscitation of 10–15% of all patients who survived OHCA in Ireland [5]. Traditionally GPs have encountered OHCA only during routine activities. Since 2015, a pilot project (‘MERIT 3’) has allowed emergency medical services (EMS) to alert volunteer GPs to nearby cardiac arrest using real-time text message technology. This project is thought to be the first of its kind. To date over 150 GPs have volunteered to participate and in the project’s first year 51 incidents of OHCA were attended [11].

The contribution of GPs dispatched to OHCA by the EMS is potentially significant. Such roles are novel and, if sustainable, must be compatible with services provided by GPs. Given the voluntary nature of participation, issues around recruitment and the factors that motivate participation are important. Furthermore, in order to develop and refine the project, it is necessary to gain an understanding of the realities of the care provided and the potential challenges faced by participants.

The objectives of this study are to explore the reasons why GPs volunteer to be alerted, and respond to OHCA in the community, and to understand GPs’ experiences in providing this care.

## Methods

A qualitative study, utilizing in-depth semi-structured interviews followed by thematic analysis was undertaken. The methodological approach was informed by the standards for reporting qualitative research outlined by O’Brien et al. [12] and the analytic approach outlined by Braun and Clarke [13]. The type of qualitative approach employed was primarily phenomenological; the research process focussed on understanding the participant’s view of self and their surrounding world in the specific context of OHCA response [14]. Steps to ensure credibility in the analysis process were conducted. Ethical approval was granted by the UCD Human Research Ethics Committee (Ref LS-17-42-Barry).

### Participants

Interviews were conducted with 14 GPs (12 male, 2 female) who had experience of responding to cardiac arrest as part of the MERIT 3 project. Participants varied in age and were all actively practising GPs.

### Recruitment

A purposive approach to sampling was undertaken; all enrolled MERIT 3 GPs who had experience of responding to cardiac arrest at the time of the study were identified (*n* = 33) and invited to participate via postal invitation in August 2017. A study information leaflet was included in the initial mailing. Twenty-seven GPs (82%) indicated interest and of this, 14 GPs were selected to participate based on achieving a spread of geographic work contexts. The number of interviews conducted balanced the requirement for rich data acquisition with the pragmatic issues of conducting multiple interviews at different geographical locations.

### Data collection

One researcher (TB) conducted all interviews, which were face-to-face and took place between September 2017 and December 2017. (In total, 13 interviews were conducted as two GPs elected to be interviewed together.) Prior to conducting any interviews, authors TB and SG (an experienced qualitative researcher) formally planned the interview process; potential biases were considered via a reflective process and a formal reflexivity statement was composed. The researcher (TB) aimed to identify any personal biases that might affect the research process or results.

The observation that the researcher came from the ‘world view perspective’ of a GP who himself participated in cardiac arrest response was considered in detail at this stage.

A semi-structured interview format was undertaken to allow flexibility of response and follow-up ‘unplanned’ issues to facilitate a participant led exploration of topics [14]. This approach involved a pre-prepared interview guide/schedule to facilitate overall structure but did not mandate rigid adherence to precise wording of questions or the order in which they were asked [15]. The written interview schedule considered two key domains:

1. The participant’s motivation to participate in the scheme
2. The realities and challenges of the process of responding to cardiac arrest in the community.

Each domain involved standard initial open-ended prompt questions and flexible follow-on questions based on the interviewee’s initial responses.

Informed written and verbal consent were obtained before commencing each interview. The initial interview acted as a pilot where the candidate was asked to give feedback and suggestions regarding the interview process. Immediately following each interview reflections concerning the interview process and content were also recorded as field notes in a research diary. All interviews were audio recorded using a Zoom H2n professional grade recorder and later professionally transcribed. The transcripts were then checked against the tapes for accuracy and minor corrections were made as necessary.

### Data analysis

Data were analysed using the thematic analysis (TA) framework outlined by Braun and Clarke [13]. TA is a method for identifying, analysing and reporting themes within data. It involves six phases; ‘data familiarization’, ‘generating initial codes’, ‘searching for themes’, ‘reviewing themes’, ‘defining and naming themes’ and ‘producing a report’. TA was selected based on its accessibility as a qualitative research approach and its flexibility in terms of theoretical framework [15]. Such flexibility was considered necessary to facilitate a level of bottom-up inductive analysis of the participant data, in the context of a researcher who possessed an inherent disciplinary standpoint as a GP who responds to OHCA. Initial coding and all further phases of TA were conducted using NVivo 11 for Windows. An experienced qualitative researcher (SG) acted as co-researcher at all stages of the analytic process to ensure credibility. SG coded a sample of the dataset (one interview) independently and reviewed all candidate and final worked out themes with TB thus supporting a reflexive process throughout the data analysis. The analysis produced a structure of themes in relation to key domains that reflect the focus of the study.

## Results

### Domain 1: Motivation to participate

Key themes and sub-themes considering the domain of ‘motivation to participate’ are reported in Tables 1 and 2.

**Table 1.** Motivation to participate; key themes and sub-themes.

Theme/sub-theme	Description	Illustrative quote(s)
Participation is a function of GPs relationship to community	Participation is a function of how GPs perceive their relationship to the community.	GP7: I’m here thirty-something years, so I actually don’t like ever refusing, if you’re the local GP. Because I know, if on a Sunday morning my car is flat, I can go up to your man and say come down and fix the car.
Obligation	GPs can perceive a sense of professional responsibility to provide this type of care.	GP12: I mean … I have the training, I have the knowledge. So I feel there is maybe a responsibility on me to do what I can do to help. Because of being a doctor really. Just, you know, it’s kind of your job. GP14: I do feel a need. And I do feel, I get this call, I get a guilt. If I can’t go, I get a guilt.
GPs are embedded in community	GPs identify themselves as a component of the community.	GP4: I’m in a situation where I live in the community and in my time I’ve been in the vast majority of the houses in the middle of the night and that obviously has a knock-on effect. I think you feel more part of the community and you feel, you know, you have I suppose a certain position within the community.
The changing nature of general practice may be a threat to participation	The nature of GP is perceived as changing; increasing professional demands, recruitment issues, changing expectation of work–life boundaries and the feminization of general practice may challenge participation.	GP14: General practice has changed even in my twenty years in general practice…. The demand service is higher, the administration work around it is higher, the medications are more complex…. And also people’s lives are busier…. And there is a lot of change in the sea where there’s feminization of general practice, has been quite noticeable in the last number of years. And they have other commitments and it is very difficult to be female, have a home life, and be a GP.
		GP12: The role of general practice has changed a lot. Because when I was here first I was called to every emergency…. Now I’m not because for, you know, road traffic collisions—it’s ambulance service, fire brigade. We don’t get called. The out-of-hours service they are urgent out of hours, not emergency. So you don’t get called to the cardiac arrests. You don’t get called.

**Table 2.** Motivation to participate; key themes and sub-themes (cont.).

Theme/sub-theme	Description	Illustrative quote(s)
Participatory gains	GPs derive specific gains from participation including maintaining a connection with an interesting and important area of practice.	GP9: Whenever there’s an emergency it tends to be me that gets sent out and the others will hold the fort…. Okay, so I mean it’s an area I’m interested in, I enjoy it, especially say working with, I enjoy the interactions we’ll say with the ambulance crew .
Maintaining emergency interest and preparedness	Many GPs had prior experience of and interest in emergency care which they were keen to maintain.	GP13: I think it’s a skill set that if you have it you should use it effectively. And you should obviously continue to use it, otherwise you’ll lose it. It’s interesting, you’re up skilling. You’re hopefully keeping your skills up, hopefully relearning.
Making a difference	GPs perceived this type of clinical activity to be satisfying and of value.	GP3: And then you know obviously as I say a few of the calls have been my own patients so it’s, you know, it’s good to be there. I mean you’re going to be involved at some stage. You might as well be there and kind of giving the person the best chance possible.… In terms of, we’ll say, you know, the ones that have been my patients, I think it has been beneficial to how, you know, dealing with them from there on. It has been of benefit to how that interaction has gone from there and, you know, working with them, you know, with their own issues and problems after that.
The ability to balance competing demands	Competing demands including time, financial and patient expectation must be negotiated to facilitate participation. The flexibility of the scheme, the observation that the notifications are occasional and the support of colleagues and family facilitate participation.	GP8: Yeah, I mean it’s … that’s general practice, it’s just a queue of people and you have to take a gap or take a rest between each one. Clear the head and go onto the next case…. And those people don’t know about the cardiac arrest or … they may not even care, so, you know … that’s people. GP9: My wife is very supportive so I don’t think so. If it doesn’t suit, if I’m looking after my children and there’s no one else around well that’s fine, I just can’t go, you know. GP3: From the practical side of things in terms of you coming back and not having 12 people waiting for you that, you know, you maybe have a handful but that they’ve tried to fight the fire for you in the meantime. Very practical stuff like that. Up to the point of, you know, making sure you’ve had a sandwich for your lunch or a cup of tea, you know. And that stuff is, yeah, hugely important, you know.

### Participation is a function of GPs relationship to community

GP participation in MERIT 3 appeared to be a function of a close, complex and at times symbiotic, relationship to the community. There was an altruistic desire to give back to the community but also a sense of obligation to participate expressed. GPs identified themselves both personally and professionally as a component of the community. Many highlighted the potential for early OHCA care, given their presence, coupled with the fact that they possessed a relevant skill set that could be required on a time critical basis. In addition, many GPs perceived a prolonged ambulance response to their locality. For some GPs, there was also a sense that regardless of the MERIT 3 scheme they would likely be summoned to participate in OHCA care.

Some GPs cautioned of the changing nature of general practice and its relationship to the community. One change highlighted was a current perceived difficulty in attracting GPs to rural areas. General practice was perceived to have become increasingly demanding. Overall morale was considered by some to be low. The feminization of general practice was highlighted as a potential challenge, as was a perceived increased focus on work–life balance and sessional commitment amongst younger GPs. Improvements in the ambulance service had meant that some ‘emergency work’ was no longer considered routine general practice.

### Participatory gains

GPs reported deriving specific positive gains from participating in the programme. The programme allowed GPs to maintain a connection with an interesting area of practice. Many of the GP participants had both prior experience of and interest in emergency care. Participation in MERIT 3 was considered a means of keeping important emergency care skills up to date. GPs reported that by responding to OHCA they had the potential to make a difference to their communities. This could be in terms of patient survival but could also involve the provision of compassionate care to families and bystanders. Such care was noted to strengthen ongoing relationships with patients and the community.

### The ability to balance competing demands

GPs highlighted many and varied competing demands that must be negotiated to facilitate participation. Specific barriers included time and financial pressures. Some GPs highlighted that not all patients might be sympathetic to the GPs involvement in OHCA response. GPs also detailed various facilitators. The voluntary nature of participation created flexibility. The perception that the alerts were intermittent and thus of a manageable workload was also highlighted. GPs who worked in group practices noted how their colleagues supported their involvement by participating in the response or covering the GP’s workload while they were responding. Participant GPs described support from their families, however, participation was recognized as having the potential to cause family friction.

### Domain 2: the realities and challenges of voluntary community cardiac arrest response

Key themes and sub-themes considering the domain of ‘the realities and challenges of voluntary community cardiac arrest response’ are reported in Tables 3–5.

**Table 3.** Realities and challenges of voluntary community cardiac arrest response; key themes and sub-themes.

Theme/sub-theme	Description	Illustrative quote(s)
Key procedural elements of response	GPs highlighted key procedural elements of the MERIT 3 scheme.	
Kit provided is useful but hard to replace	GPs highlighted that the kit provided by the scheme was useful, however replacing it once used was an issue.	GP11: I am not sure how we replace stuff, that is one thing that I am not too clear on. You know at the moment it has been a bit by grace and favour of the ambulance guys and if you know them. I suppose if there was a more formal method or if you knew, look I have used this stuff, this is where I go to get it, or maybe we’re supposed to get it ourselves? It is not quite sure.
Technology can act both as a help and a hindrance	Many aspects of participation relied on useful technology. Occasionally there was frustration with some aspects of the relevant technological systems.	GP2: As far as the alert system yeah, I think it is perfect, you have your phone in your pocket all the time. GP4: It does get annoying, because at this stage I get, sometimes I get five or six texts about the one call.
Driving has risk, but safety is a priority	In general GPs considered safe driving to be a necessary priority despite a sense of urgency to respond.	GP1: You do feel pressurised, I do feel pressurised, the last one that I was at … I thought I was taking a short-cut but I got caught in traffic you know it is pressurised, I'm very careful, mindful that I'm not licenced to drive beyond the speed limit, I'm not licenced to drive in any way dangerously.

**Table 4.** Realities and challenges of voluntary community cardiac arrest response; key themes and sub-themes (cont.).

Theme/sub-theme	Description	Illustrative quote(s)
The GP role in OHCA response is multifaceted	GPs described a spectrum of different clinical roles when responding to OHCA.	GP10: you’ll confer with whosoever is there you know, and it could be the ambulance service, it could be the guards (police). You know I've been involved where it's been more a forensic exercise with the guards and so you establish what needs to be done. Your role as a GP would cover from first on scene to providing support as part of a resuscitation team to, it's still the legal role of the doctor to pronounce a patient dead even though resuscitation may have been ceased before you arrived and then the conferring with the coroner, conferring with the guards, providing emotional support to the family, so all those roles.
Filling a gap	Providing early basic life support treatment was identified as a key component of care and was considered especially important where ambulance response might be delayed.	GP12: Average (ambulance) response time is probably close to 40 (min) and it’s not infrequently that it’s over an hour. So in that first period of time, whatever we can do for them is as much as is gonna be done. GP5: And so my idea of turning up would only be to fill in until they (paramedics) arrive and then I would stand back.
Rowing in with paramedics but at times providing a 'broader' perspective	GPs considered paramedics to be very skilled in OHCA resuscitation and were generally happy to 'fit in' with their care plan. GPs did recognize that occasionally the 'broader perspective' brought by the GP could be useful. In particular, GPs identified decision-making around resuscitation termination to be a key aspect of GP care.	GP11: Paramedics and advanced paramedics are so used to doing this sort of thing as well that often you find that you sort of fall into a role depending on what is happening and what is going on. GP12: They have a narrow skill set but they’re very, very good with that narrow skill set. They’re much better at doing it than we are because they’re doing it much more often. The only difference is that we’re looking at a problem from first principles whereas they’re looking at a problem in terms of the protocol that they want to apply. That’s the only difference. And occasionally there are situations where you’re going to use something or a technique which just isn’t in the CPGs (clinical practice guidelines) GP13: It’s a difficult decision for anyone to make (ceasing resuscitation). They’re following a CPG guideline. I suppose I’m looking at it slightly more holistically as a GP, do you know ‘Is it worth giving this another shot or is this, look, we’ve gone to the notes, he’s got a history…’ and perhaps it’s, it’s still very clinical, but it’s taking maybe a slightly different approach to it.
Caring for bystanders and families	GPs described a specific and significant aspect of their role in OHCA care as ‘caring for families’.	GP14: But you are a GP and you have to pick up the pieces to make things easier for a family. If I didn’t respond what would happen? They’d move the person and that would be it. There’s nobody left with the family. So I do think there is an important role there. GP13: I do an aftercare visit, you know, down the line, maybe a month after, if the whole family are my patients. If they’re not, in my practice we send a mass card and if they’re somebody I’ve looked after for a long time we would try and attend the funeral.

**Table 5.** Realities and challenges of voluntary community cardiac arrest response; key themes and sub-themes (cont.).

Theme/sub-theme	Description	Illustrative quote(s)
Difficult situations with potential for psychosocial impact	GPs highlighted the complex situations and potential psychosocial burden that could accompany participation.	GP7: Oh well, oh my god, the whole place ran, there was screaming and roaring all over the place, it was horrific. But, did it affect me personally? Yeah, that day it did, it was horrific. GP2: There might be pressure from the family pro or anti-resuscitation, there might be pressure from the nursing home to transport the patient out, there might be pressure from your colleagues. So, I have been involved in resuscitations where I wasn’t fully comfortable with resuscitating the patient to start with and I have just been, not bullied but I have been put under a lot of pressure to continue resuscitation where I probably wasn’t that comfortable with it.

### Key procedural elements of response

GPs discussed the key complexities of the process of an alert and response and highlighted several specific issues. In general, the equipment provided was considered a positive aspect of the scheme; however, replacing consumables was highlighted as a specific issue.

Participants described a multitude of interactions with technology around the process of OHCA response. Some aspects were seen as enhancing OHCA response, however, others were seen as unhelpful. GPs reported that they found the text message system to work well but did report some frustration in the observation that a single incident often involved several text message alerts. One GP highlighted this as a potential safety issue while driving. GPs often described using smartphone technology to locate patients. This was considered an efficient way of navigating to the OHCA location. Some GPs reported that newly assigned post codes (Eircodes) had been of additional assistance. GPs described routinely contacting the ambulance control dedicated phone line and felt this was an important logistic element of the response with regard to locating the patient.

The issue of driving to an emergency was specifically explored. Most GPs highlighted the need for considered safe driving under what could be stressful circumstances. Some GPs did feel that the pressure of the response situation could lead to risky driving behaviours. Others highlighted that responding to emergencies in a car was a routine component of GP care.

### The GP role in OHCA response is multifaceted

GPs highlighted a multifaceted role when providing OHCA care ranging from early basic life-support care, assisting paramedics, offering a broader medical perspective that contributed to decision-making and providing information and psychosocial support to families both during and after the resuscitation effort.

Participant GPs highlighted situations where the ambulance response to OHCA would be prolonged and they described a key role in providing early care. GPs perceived paramedics to be highly skilled clinicians in the area of OHCA resuscitation. They recognized that paramedics were exposed to resuscitation more frequently than GPs and in general were happy to defer to paramedics in running the resuscitation algorithm once they arrived on scene. GPs reported their interactions with paramedics to be largely very positive. They noted that paramedics were operating within the framework of a fixed care algorithm, while the GP was operating from a more flexible framework of ‘first principles’. Some GPs did report that occasionally there could be friction between paramedics and GPs; occasionally individual GPs had felt undermined by the actions of paramedics. This could have related to a conflict around overall responsibility for a patient’s care or a clash between a protocol-based approach and one of broader clinical reasoning.

By virtue of their medical training and position in the community, GPs highlighted that they could possess extra knowledge that might inform decision-making in OHCA care. This was perceived to have particular relevance in the decision to continue or cease resuscitation. Although this decision was frequently considered to be a ‘team decision’ GPs perceived significant overall personal responsibility. Participant GPs described a specific and significant aspect of their role in OHCA care as ‘caring for families’. GPs highlighted a sense of continuity about this care that went beyond the actual resuscitation effort through to aftercare for patient and family as relevant. GPs considered the ‘caring for families’ role as an important and integral part of the overall GP OHCA skill set. GPs felt well equipped for this care and for some this was considered a skill set more specific to GPs than paramedics.

### Difficult situations with potential for psychosocial impact

GPs highlighted the potential psychosocial burden that could accompany participation in MERIT 3. Specific circumstances were considered to be of greater risk including situations involving children, suicides and situations where the resuscitation team interactions had been difficult. The observation that a GP’s contribution to patient care might not always be perceived positively was highlighted by some. Other GPs highlighted a physiological ‘adrenaline rush’ that accompanies responding to OHCA and the resultant challenge in returning to normal activities in the aftermath. At times some GPs described a sense of trepidation or performance anxiety around providing OHCA care. OHCA care was considered an infrequent but high stakes aspect of general practice. In addition, it was recognized that complex decision-making could, at times, result in internal conflict. GPs reported that they learn to cope with such psychosocial impact via their routine role and experience as GPs. They did, however, appear to be aware of the need for ‘self-care’ around these issues. Some GPs highlighted the lack of any formal MERIT 3 support in terms of psychosocial wellbeing.

## Discussion

### Main findings

The results of this study suggest that GP participation in voluntary OHCA response can be understood as a function of GPs’ relationship to the community, and the fact that GPs derive participatory gains and the ability of GPs to manage competing demands in their personal and professional lives. In this study, GPs both expressed an altruistic desire to give back to the community and perceived a sense of obligation to participate in OHCA care. This was especially so for those who perceived their community to be remote from ambulance service resources. Some GPs cautioned of ongoing changes in general practice that could threaten participation.

GPs highlighted a complex, multifaceted role in providing OHCA community response. They derived an inherent sense of satisfaction in offering potentially life-saving care but also in the provision of holistic, compassionate end-of-life care for patients and their families. GPs considered paramedics to be expert in OHCA resuscitation, however most also perceived an inherent value in supplementing the paramedic approach with a somewhat broader GP perspective. Participation in OHCA response was not without psychological risk for GPs, however such risks were considered to mirror those of many ‘routine’ day to day GP activities. The voluntary nature of participation, the intermittent and infrequent nature of alerts as well as the support from the GP’s own family and colleagues ultimately allowed participants to integrate OHCA response with the demands of personal and professional life.

### Strengths and limitations

Our study has a number of strengths that support its ability to inform understanding, including the use of published guidelines in the development and implementation of the research, the positive role of the researcher’s own experience of the topic and the collaboration with an experienced qualitative researcher. As far as we are aware this is the first study to explore the reasons why GPs volunteer to be cardiac arrest first responders and also explore what such a role involves. The results have the potential to stimulate and inform similar initiatives in other countries.

There are important limitations that should be considered in interpreting the results of this study. Although the qualitative semi-structured interview design was useful in facilitating an in-depth exploration of GP perspectives, the lack of standardization of this approach can raise concerns about reliability and the potential for biases [14]. One researcher (TB) conducted all interviews and led the data analysis. As this researcher possesses an inherent disciplinary standpoint as a GP who responds to OHCA, the possibility that this perspective could have created bias in the dialogue or content analysis must be considered. An active reflective process throughout the study facilitated by the involvement of a second researcher (SG) aimed to limit this potential.

The themes and issues raised offer significant insight into the motivation of and challenges faced by GPs who participate in OHCA voluntary first response. The qualitative design with small absolute numbers of participants cannot however assume generalizability but rather it should be seen as offering hypothesis generating insights into what is a complex phenomenon.

### Comparison with existing literature

There are limited existing data available considering this topic area. Previous research from Singapore suggested that over 60% of primary healthcare doctors surveyed were willing to participate in a GP defibrillation project [16]. Other research from the UK found that over half of GPs in one region would be willing to participate in a rapid response cardiac arrest scheme [17]. The findings of our study have similarity with research from Norway that found that though the role of rural GP emergency care had changed over time, rural GPs considered emergency care to remain an important aspect of their practice and to be of value to their patients [18]. Furthermore, as in our study, participation in emergency care was perceived to promote increased GP emergency care skills and competency [18].

### Implications

GPs are willing to volunteer for OHCA community emergency first response. Where GPs can reach patients quickly additional lives can be saved. GP first responders also facilitate the provision of compassionate, holistic care to patients and their families during and in the aftermath of OHCA. Such holistic care is likely to have significant overall importance given that the majority of patients for whom an EMS cardiac arrest response is generated ultimately do not survive [19]. Although both GP roles in OHCA resuscitation and in OHCA end-of-life care have previously been highlighted, this research provides the first in-depth exploration of such care [3,4]. The nuanced nature of this care delivered alongside existing general practice roles, places it within the domain of personalized medicine routinely offered in general practice [20].

To some extent, all of the care already considered depends on GPs’ willingness to be present in and available to their communities. There has been significant recent discussion of the pressures faced by general practice [21,22]. Despite such pressures, it is notable that many GPs in Ireland are prepared to volunteer for the role of OHCA first responder. Changing work practices and a move toward sessional work commitments may threaten GP involvement in OHCA emergency care. Alternatively, initiatives that sustain and build capacity in general practice could have positive effects on the early availability of community emergency care.

Finally, the provision of support for GPs who participate in voluntary OHCA response warrants further consideration. Previous research has highlighted the psychological impact of participation in cardiac arrest response among other disciplines and individuals [23–25]. Some studies suggest the incidence of significant prolonged adverse effects are likely to be low [26,27]. Others suggest a risk of long-term emotional and social morbidity [28–30]. GPs may not be immune to these risks.

## Conclusion

GP first responders provide holistic cardiac arrest care that includes basic life-support, team working with paramedics, critical decision-making and the provision of psychosocial support for bystanders and family members. GPs volunteer for this role because of their relationship to the community.

## References

[1] Atwood C, Eisenberg MS, Herlitz J, et al. Incidence of EMS-treated out-of-hospital cardiac arrest in Europe. Resuscitation. 2005;67(1):75–80. 16199289 10.1016/j.resuscitation.2005.03.021

[2] Hasselqvist-Ax I, Riva G, Herlitz J, et al. Early cardiopulmonary resuscitation in out-of-hospital cardiac arrest. N Engl J Med. 2015;372(24):2307–2315. 26061835 10.1056/NEJMoa1405796

[3] Colquhoun M. Resuscitation by primary care doctors. Resuscitation. 2006;70(2):229–237. 16814447 10.1016/j.resuscitation.2006.01.007

[4] Masterson S, Vellinga A, Wright P, et al. General practitioner contribution to out-of-hospital cardiac arrest outcome: a national registry study. Eur J Gen Pract. 2015;21(2):1–137. 10.3109/13814788.2014.962509 25387228

[5] Barry T, Headon M, Glynn R, et al. Ten years of cardiac arrest resuscitation in Irish general practice. Resuscitation. 2018;126:43–48. 29510194 10.1016/j.resuscitation.2018.02.030

[6] OHCAR. Out of Hospital Cardiac Arrest Register Ireland; Annual Report 2016.

[7] OHCAR. Out of Hospital Cardiac Arrest Register Ireland; Annual Report 2017.

[8] Teljeur C, Tyrrell E, Kelly A, et al. Getting a handle on the general practice workforce in Ireland. Ir J Med Sci. 2014;183(2):207–213. 23900944 10.1007/s11845-013-0991-1

[9] Bury G, Headon M, Dixon M, et al. Cardiac arrest in Irish general practice: an observational study from 426 general practices. Resuscitation. 2009;80(11):1244–1247. 19720440 10.1016/j.resuscitation.2009.07.010

[10] Bury G, Egan M, Tobin H, et al. Immediate care training in Ireland, 2002-2013: a potential link between high uptake rates and effect. Ir Med J. 2015;108(5):140. 26062239

[11] Barry T, Conroy N, Headon M, et al. The MERIT 3 project: alerting general practitioners to cardiac arrest in the community. Resuscitation. 2017;121:141–146. 29097197 10.1016/j.resuscitation.2017.10.025

[12] O’Brien BC, Harris IB, Beckman TJ, et al. Standards for reporting qualitative research: a synthesis of recommendations. Acad Med. 2014;89(9):1245–1251. 24979285 10.1097/ACM.0000000000000388

[13] Braun V, Clarke V. Using thematic analysis in psychology. Qual Res Psychol. 2006;3(2):77–101.

[14] Robson C. Real world research: a resource for users of social research methods in applied settings. 3rd ed. Oxford: Wiley-Blackwell; 2011.

[15] Braun V, Clarke V. Successful qualitative research: a practical guide for beginners. London: Sage; 2013.

[16] Ong MEH, Chan YH, Ang HY, et al. Resuscitation of out-of-hospital cardiac arrest by Asian primary health-care physicians. Resuscitation. 2005;65(2):191–195. 15866400 10.1016/j.resuscitation.2004.11.021

[17] Soo L, Smith N, Gray D. The place of general practitioners in the management of out-of-hospital cardiopulmonary resuscitation. Resuscitation. 1999;43(1):57–63. 10636318 10.1016/s0300-9572(99)00123-9

[18] Hjortdahl M, Halvorsen P, Risor MB. Rural GPs' attitudes toward participating in emergency medicine: a qualitative study. Scand J Prim Health Care. 2016;34(4):377–384. 27827547 10.1080/02813432.2016.1249047 PMC5217286

[19] Dyson K, Brown SP, May S, et al. International variation in survival after out-of-hospital cardiac arrest: a validation study of the Utstein template. Resuscitation. 2019;138:168–181. 30898569 10.1016/j.resuscitation.2019.03.018

[20] Maier M. Personalized medicine-a tradition in general practice. Eur J Gen Pract. 2019;25(2):63–64. 31032696 10.1080/13814788.2019.1589806 PMC6495112

[21] Wilkie V, Ralphs A. The pressures on general practice. BMJ. 2016;353:i2580. 27169432 10.1136/bmj.i2580

[22] Thompson M, Walter F. Increases in general practice workload in England. Lancet. 2016;387(10035):2270–2272. 27059885 10.1016/S0140-6736(16)00743-1

[23] Phung VH, Trueman I, Togher F, et al. Community first responders and responder schemes in the United Kingdom: systematic scoping review. Scand J Trauma Resusc Emerg Med. 2017;25(1):58. 28629382 10.1186/s13049-017-0403-z PMC5477292

[24] Phung VH, Trueman I, Togher F, et al. Perceptions and experiences of community first responders on their role and relationships: qualitative interview study. Scand J Trauma Resus Emerg Med. 2018; 526(1):13. 10.1186/s13049-018-0482-5 PMC5800091 29402312

[25] Harrison-Paul R, Timmons S, Schalkwyk W. D V. Training lay-people to use automatic external defibrillators: are all of their needs being met? Resuscitation. 2006;71(1):80–88. 16945471 10.1016/j.resuscitation.2006.02.008

[26] Zijlstra JA, Beesems SG, De Haan RJ, et al. Psychological impact on dispatched local lay rescuers performing bystander cardiopulmonary resuscitation. Resuscitation. 2015;92:115–121. 25957944 10.1016/j.resuscitation.2015.04.028

[27] Peberdy MA, Ottingham LV, Groh WJ, et al. Adverse events associated with lay emergency response programs: the public access defibrillation trial experience. Resuscitation. 2006;70(1):59–65. 16784998 10.1016/j.resuscitation.2005.10.030

[28] Mathiesen WT, Bjørshol CA, Braut GS, et al. Reactions and coping strategies in lay rescuers who have provided CPR to out-of-hospital cardiac arrest victims: a qualitative study. BMJ Open. 2016;6(5):e010671. 10.1136/bmjopen-2015-010671 PMC4885284 27225648

[29] Genest M, Levine J, Ramsden V, et al. The impact of providing help: emergency workers and cardiopulmonary resuscitation attempts. J Trauma Stress. 1990;3(2):305–313.

[30] Axelsson Å, Herlitz J, Karlsson T, et al. Factors surrounding cardiopulmonary resuscitation influencing bystanders' psychological reactions. Resuscitation. 1998;37(1):13–20. 9667333 10.1016/s0300-9572(98)00027-6

